# Supplementary material for: Genetic Concordance in Primary Cutaneous Melanoma and Matched Metastasis: A Systematic Review and Meta-Analysis
Source: Int J Mol Sci. 2023 Nov 14;24(22):16281. doi: 10.3390/ijms242216281 (PMC10671327; doi:10.3390/ijms242216281)
Supplement: Supplementary file 1 [file ijms-24-16281-s001.zip › ijms-2699572-supplementary.pdf]

Supplementary Materials

Table S1. The search strategies used in the different databases. The asterisk * in a search strategy is used as a wildcard character to represent various word endings or variations.

Database (Number of searched studies)	Search strategy
**Embase.com (1878)**	(('metastatic melanoma'/exp OR (metastasis/de AND melanoma/de) OR ((metasta* NEAR/6 melanoma*)):ab,ti) AND ('primary melanoma'/de OR ('primary tumor'/de AND melanoma/exp) OR ((primary NEAR/6 melanoma*)):Ab,ti) OR ((melanoma* AND (primary NEAR/6 metasta*)):Ab,ti) OR ((melanoma* AND (primary AND metasta*)):ti)) AND ('mutation'/exp OR 'copy number variation'/de OR 'mutational analysis'/exp OR 'DNA sequence'/de OR 'mutational analysis'/de OR 'gene sequence'/de OR 'DNA fragment'/de OR 'genomic DNA'/de OR 'polymerase chain reaction'/exp OR 'sequence analysis'/exp OR 'molecular pathology'/de OR 'genotyping technique'/exp OR 'nucleotide sequence'/de OR 'promoter region'/de OR 'molecular fingerprinting'/exp OR 'tumor-related gene'/exp OR 'clonal variation'/de OR 'genetic association'/de OR 'molecular genetics'/de OR (sequenc* OR ((DNA OR deoxyribonucl* OR genom* OR gene OR genes OR genetic*) NEAR/3 (alterat* OR characteri* OR fragment* OR associat*)) OR (dna NEAR/3 (genom*)) OR polymerase-chain-reaction OR pcr OR qpcr OR rtpcr OR (mutation* NEAR/6 (analy* OR profil*)) OR (molecul* NEAR/3 (patholog* OR pathogenes* OR fingerprint* OR finger-print* OR profile* OR classificat* OR genetic*)) OR (genotyp* NEAR/3 (technique* OR assign* OR determin* OR assay*)) OR promoter-region* OR oncogene* OR BRAF OR NRAS OR PTEF OR ((gene OR genes) NEAR/3 (tumor* OR tumour*)) OR mutation* OR mutant* OR mutagenes* OR deletion* OR copy-number-varia* OR (clonal NEAR/3 variation*) OR clonalit*):ab,ti) NOT ('cancer of unknown primary site'/mj OR (unknown* NEAR/3 primar*):ti) NOT (([Conference Abstract]/lim AND [1800-2020]/py) OR [Letter]/lim OR [Note]/lim OR [Editorial]/lim) AND [english]/lim NOT ([animals]/lim NOT [humans]/lim)
**Medline ALL Ovid (1451)**	(((Neoplasm Metastasis / AND Melanoma /) OR ((metasta* ADJ6 melanoma*)).ab,ti.) AND (((primary ADJ6 melanoma*)).ab,ti.) OR ((melanoma* AND (primary ADJ6 metasta*)).ab,ti.) OR ((melanoma* AND (primary AND metasta*)).ti.)) AND (Mutation/ OR DNA Copy Number Variations/ OR DNA Mutational Analysis/ OR Base Sequence/ OR Polymerase Chain Reaction/ OR exp Sequence Analysis/ OR Pathology, Molecular/ OR Genotyping Techniques/ OR Promoter Regions, Genetic/ OR DNA Fingerprinting/ OR Genetic Association Studies/ OR Molecular Biology/ OR (sequenc* OR ((DNA OR deoxyribonucl* OR genom* OR gene OR genes OR genetic*) ADJ3 (alterat* OR characteri* OR fragment* OR

Database (Number of searched studies)	Search strategy
	associat*)) OR (dna ADJ3 (genom*)) OR polymerase-chain-reaction OR pcr OR qpcr OR rtpcr OR (mutation* ADJ6 (analy* OR profil*)) OR (molecul* ADJ3 (patholog* OR pathogenes* OR fingerprint* OR finger-print* OR profile* OR classificat* OR genetic*)) OR (genotyp* ADJ3 (technique* OR assign* OR determin* OR assay*)) OR promoter-region* OR oncogene* OR BRAF OR NRAS OR PTEF OR ((gene OR genes) ADJ3 (tumor* OR tumour*)) OR mutation* OR mutant* OR mutagenes* OR deletion* OR copy-number-varia* OR (clonal ADJ3 variation*) OR clonalit*).ab,ti.) NOT (*Neoplasms, Unknown Primary/ OR (unknown* ADJ3 primar*).ti.) AND english.la. NOT (exp animals/ NOT humans/)
**Web of Science (1632)**	TS=(((((metasta* NEAR/5 melanoma*))) AND (((primary NEAR/5 melanoma*))) OR ((melanoma* AND (primary NEAR/5 metasta*)))) AND ((sequenc* OR ((DNA OR deoxyribonucl* OR genom* OR gene OR genes OR genetic*) NEAR/2 (alterat* OR characteri* OR fragment* OR associat*)) OR (dna NEAR/2 (genom*)) OR polymerase-chain-reaction OR pcr OR qpcr OR rtpcr OR (mutation* NEAR/5 (analy* OR profil*)) OR (molecul* NEAR/2 (patholog* OR pathogenes* OR fingerprint* OR finger-print* OR profile* OR classificat* OR genetic*)) OR (genotyp* NEAR/2 (technique* OR assign* OR determin* OR assay*)) OR promoter-region* OR oncogene* OR BRAF OR NRAS OR PTEF OR ((gene OR genes) NEAR/2 (tumor* OR tumour*)) OR mutation* OR mutant* OR mutagenes* OR deletion* OR copy-number-varia* OR (clonal NEAR/2 variation*) OR clonalit*))) NOT ti=((unknown* NEAR/2 primar*)) and DT=(article) AND LA=(english)
**Cochrane CENTRAL Register of Trials (356)**	((((metasta* NEAR/6 melanoma*)):ab,ti) AND (((primary NEAR/6 melanoma*)):Ab,ti) OR ((melanoma* AND (primary NEAR/6 metasta*)):Ab,ti) OR ((melanoma* AND (primary AND metasta*)):ti)) AND ((sequenc* OR ((DNA OR deoxyribonucl* OR genom* OR gene OR genes OR genetic*) NEAR/3 (alterat* OR characteri* OR fragment* OR associat*)) OR (dna NEAR/3 (genom*)) OR polymerase-chain-reaction OR pcr OR qpcr OR rtpcr OR (mutation* NEAR/6 (analy* OR profil*)) OR (molecul* NEAR/3 (patholog* OR pathogenes* OR fingerprint* OR finger-print* OR profile* OR classificat* OR genetic*)) OR (genotyp* NEAR/3 (technique* OR assign* OR determin* OR assay*)) OR promoter-region* OR oncogene* OR BRAF OR NRAS OR PTEF OR ((gene OR genes) NEAR/3 (tumor* OR tumour*)) OR mutation* OR mutant* OR mutagenes* OR deletion* OR copy-number-varia* OR (clonal NEAR/3 variation*) OR clonalit*):ab,ti) NOT ((unknown* NEAR/3 primar*):ti)

The following information was used in g:Profiler as input data:

Gene list: *CDK4* *MAP2K1* *RB1* *CTNNB1* *HNFA1* *RAC1* *CDKN2A*
PIK3CA TP53 *EPHB6* *TERT* *CDKN2A* *PIK3CA* *TP53* *MAP2K1*
PTEN *CTNNB1* *HNFA1* *TP53* *MAP2K1* *PTEN* *RAC1*
BRAF *NRAS* *KIT*

Data sources: GO biological process

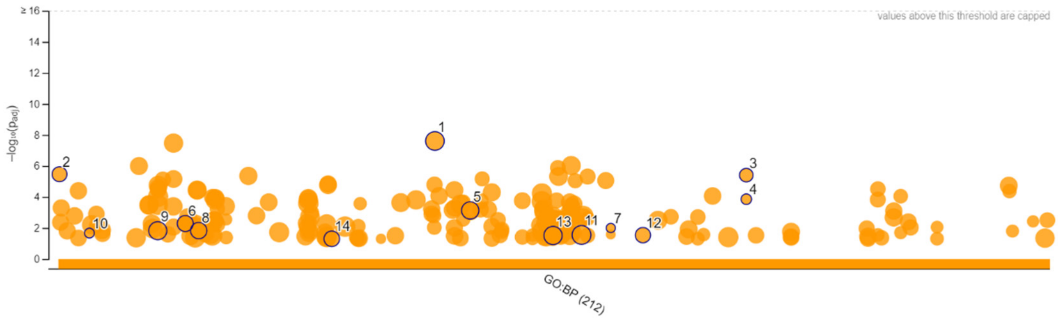

ID	Source	Term ID	Term Name	p_{adj} (query_1)
1	GO:BP	GO:0042127	regulation of cell population proliferation	2.462×10^{-8}
2	GO:BP	GO:0000082	G1/S transition of mitotic cell cycle	3.417×10^{-6}
3	GO:BP	GO:0090398	cellular senescence	3.919×10^{-6}
4	GO:BP	GO:0090399	replicative senescence	1.394×10^{-4}
5	GO:BP	GO:0044087	regulation of cellular component biogenesis	7.408×10^{-4}
6	GO:BP	GO:0009410	response to xenobiotic stimulus	5.150×10^{-3}
7	GO:BP	GO:0060440	trachea formation	1.001×10^{-2}
8	GO:BP	GO:0010035	response to inorganic substance	1.474×10^{-2}
9	GO:BP	GO:0007155	cell adhesion	1.500×10^{-2}
10	GO:BP	GO:0002318	myeloid progenitor cell differentiation	2.143×10^{-2}
11	GO:BP	GO:0051649	establishment of localization in cell	2.824×10^{-2}
12	GO:BP	GO:0061448	connective tissue development	2.909×10^{-2}
13	GO:BP	GO:0048870	cell motility	3.062×10^{-2}
14	GO:BP	GO:0031647	regulation of protein stability	4.982×10^{-2}

**version**	e110_eg57_p18_4b54a898
**date**	9/26/2023, 7:13:01 PM
**organism**	hsapiens

g:Profiler

Figure S1. Pathways analysis using g:profieler.

(**A**)

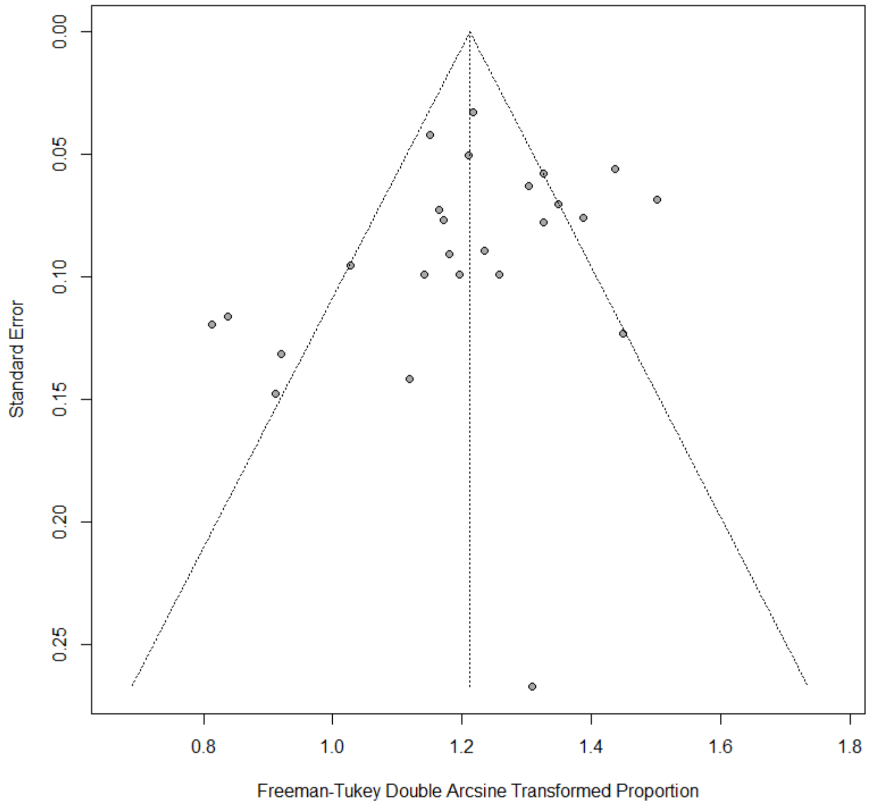

(**B**)

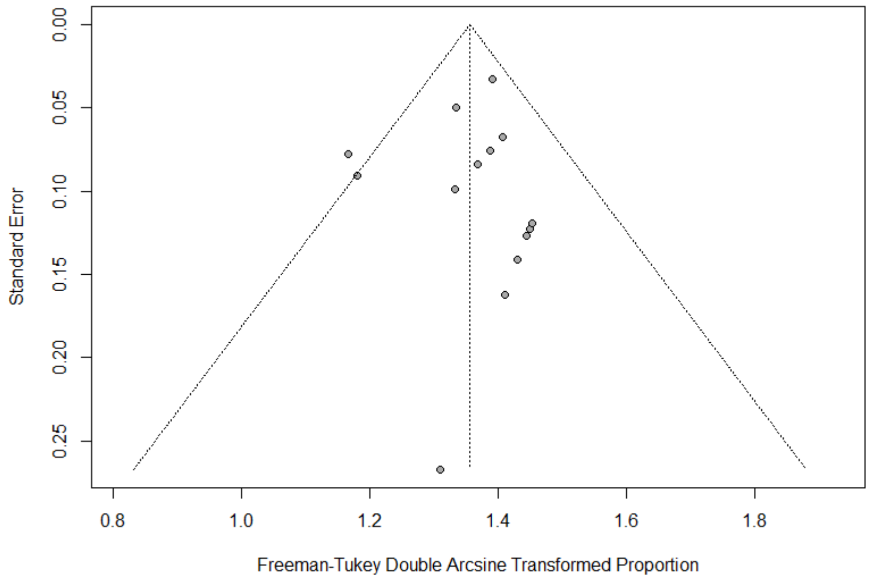

Figure S2. Study effects and publication bias were assessed using funnel plots and the Egger's test. (**A**) BRAF studies and (**B**) NRAS studies.
